# Multiple Stressor Effects of a Neonicotinoid, Heatwaves, and Elevated Temperatures on Aquatic Insect Emergence

**DOI:** 10.1021/acs.est.5c01498

**Published:** 2025-07-08

**Authors:** Markus Hermann, Mawuli K. Amekor, Enzo Contrucci, Ann M. Evarita, Edwin T.H.M. Peeters, Paul J. Van den Brink

**Affiliations:** Aquatic Ecology and Water Quality Management Group, 4508 Wageningen University & Research, P.O. Box 47, Wageningen 6700 AA, The Netherlands

**Keywords:** imidacloprid, climate change, weather extremes, combined temperature and chemical effects, insect life cycle, insect communities, mesocosm, freshwater ecosystem

## Abstract

Intensive agricultural practices, including neonicotinoid insecticides, and climate change are two potential drivers of global insect decline, contributing to biodiversity loss. However, ecologically realistic field experiments investigating these multiple stressor effects on emerging aquatic insects are scarce. To empirically test whether exposure to imidacloprid (1, 10 μg/L) and two different climate change scenarios (i) elevated temperatures (+4 °C vs. ambient temperatures) and (ii) reoccurring heatwaves (+0 to 8 °C) may cause a decline in insect emergence, we conducted an outdoor mesocosm study. Aquatic insect communities were exposed to single and combined stressors, while emergence was monitored during a 3-month period. We report significant losses in insect biomass and abundance under single and combined treatments. The high imidacloprid treatment and elevated temperatures combined caused a significant 47% decline in total insect biomass across the insect orders Diptera, Ephemeroptera, Coleoptera, Hymenoptera, Hemiptera, Odonata, and Trichoptera. Community structure and population dynamics were significantly affected, with Diptera and Ephemeroptera being most sensitive to the high and both imidacloprid treatments, respectively. Diptera dominated but was significantly reduced by the high imidacloprid and heatwave combination. Temperature-enhanced imidacloprid toxicity and the significant threat these stressors pose to aquatic insect communities highlight the need for effective climate change mitigation strategies to conserve aquatic insect biodiversity.

## Graphical abstract

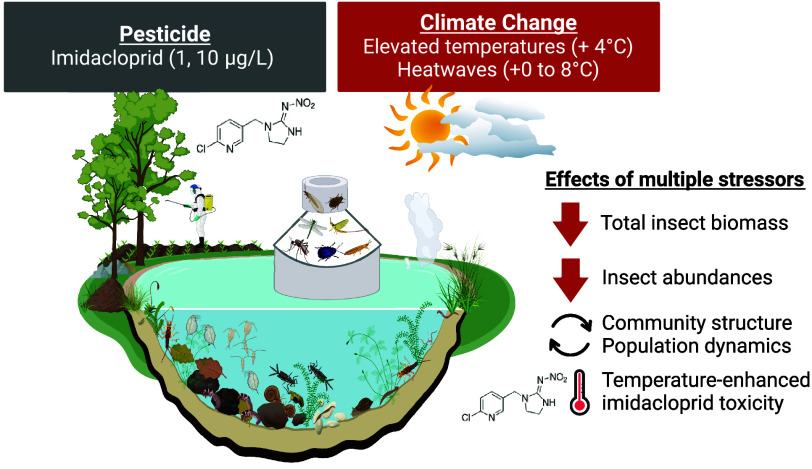

## Introduction

Insects are a unique class of animals that inhabit aquatic and terrestrial ecosystems, with millions of extant species on earth. They have lately received increased research attention in the context of agricultural activities and climate change, as these perturbations are considered major negative drivers of their biomass and community structure.^1–3^ In this context, several studies and reports consistently conveyed a core message, urgently appealing to address the global decline in both terrestrial and aquatic insects that has become apparent through reductions in species abundances, biomass, and diversity.^4–6^ Awareness exists on their pivotal role in ecosystems and the potential ecological consequences of a decrease in entomofauna biodiversity, such as the loss of freshwater ecosystem functions and services, including nutrient cycling, water purification, and pest control. These aquatic food web alterations may lead to cascading effects in terrestrial ecosystems and finally human well-being.^7–9^ There is a general lack of knowledge about why some species are declining in certain places of the world and others not.^10–12^ Sometimes there are even opposing trends in abundances or species turnover, depending on the ecosystem under investigation.^13,14^ In fact, tremendous research efforts dedicated to quantifying the potential causes of the insect declines led to various factors being regarded as main drivers of the observed trends in insect abundances, including agricultural pesticide application and climate change.^5,15–18^ However, the limited availability of comprehensive data from well-controlled, environmentally realistic aquatic and terrestrial field experiments hamper the capability to draw conclusions based on causal relationships that would support conservation efforts to counteract the broadly perceived “insect apocalypse”.^19–21^ Context-specific mesocosm experiments in the field are useful tools that enable the evaluation of single and multiple stressors in an ecologically relevant setting, thereby providing proof of the significance and relative contribution of stressors that are expected to drive these declines.^22^ In this way, mesocosm experiments can be used to reveal causal links between the stressors under investigation and the observed effects while excluding confounding factors that are always at play in long-term monitoring data.^23^

According to survey data, the increased use of insecticides, notably neonicotinoids, has been associated with decreasing insect populations as early as the introduction of these systemic pesticides designed to impair insects that are considered as pests from an agricultural standpoint.^4,24,25^ The globally most applied neonicotinoid, imidacloprid,^26^ exhibits high water solubility and low soil adsorption potential, thus posing a high risk of contaminating aquatic environments through spray-drift, runoff, and leaching.^27,28^ In fact, previous laboratory and field experiments revealed significantly negative effects of imidacloprid on aquatic nontarget organisms, including insects,^29–31^ likely attributed to its specific mode of action that targets on the postsynaptic nicotinic acetylcholine receptors of insects.^32^ Despite the European Union’s 2018 ban on imidacloprid for agricultural field purposes, environmental monitoring continues to trace concentrations ranging from approximately 2.7 ng/L to 10 μg/L.^33–36^

Freshwaters are also expected to face stressors related to global climate change, such as elevated surface temperatures and more frequent and extreme weather events like heatwaves.^37–39^ Since alterations in temperature can push species beyond their thermal tolerance range, impacting their life cycle and development,^40,41^ the combination of both chemical and temperature stress may trigger unexpected effects on the insect community and consecutive ecosystems changes.^42–45^ Albeit experimental evidence on neonicotinoid-driven aquatic insect decline exists, as shown for thiacloprid^46^ and imidacloprid,^42,47–51^ there remains a major lack of empirical knowledge about the effects of the widespread use of neonicotinoids on emerging aquatic insects, particularly under different environmentally realistic warming scenarios. The combined effects with temperature are especially relevant for imidacloprid as tropical aquatic ecosystems seem to be more sensitive to imidacloprid than temperate ones.^29,30,52^ Furthermore, prior research on the damselfly showed that environmental temperature fluctuations amplified the toxicity of the organophosphate insecticide chlorpyrifos, resulting in a 6-fold increase in mortality and reduced growth rates.^53^ This suggests that similar chemical-temperature interaction effects may also apply to the neonicotinoid insecticide imidacloprid in aquatic insect communities.

In this study, we empirically investigated the effects of environmentally relevant water concentrations of the neonicotinoid imidacloprid (1, and 10 μg/L) during a 3-month period under two different future climate warming scenarios (i.e., elevated temperatures and heatwaves) on aquatic insect emergence using 30 field pond mesocosms (Figure 1A,B). Our research hypotheses and objectives were based on recent findings on freshwater ecosystem responses to different climate-change-related temperature scenarios and chemical pollution, and were tested in this paper using an analogous experimental design.^54,55^ While we hypothesized stronger detrimental imidacloprid effects on aquatic insect emergence with increasing exposure concentrations under ambient temperatures, we expected temperature-enhanced toxicity with immediate and lasting declines in emergence under reoccurring heatwaves and delayed effects under elevated temperatures because of differences in stressor magnitude and persistence, resulting in different interaction times with biota. To address these assumptions, we assessed multiple stressor effects on emerged insect biomass and investigated taxon-specific responses by revealing emergence patterns on population and community level, considering imidacloprid’s environmental fate under the different temperature treatments. By doing so, a full-factorial experimental approach allowed us to disentangle the multiple stressor effects on emerging aquatic insects of the orders Diptera, Ephemeroptera, Coleoptera, Hymenoptera, Hemiptera, Odonata, and Trichoptera to evaluate the single and combined effects of a neonicotinoid pesticide and climate warming as potential drivers of the globally observed insect declines.

**Figure 1:**
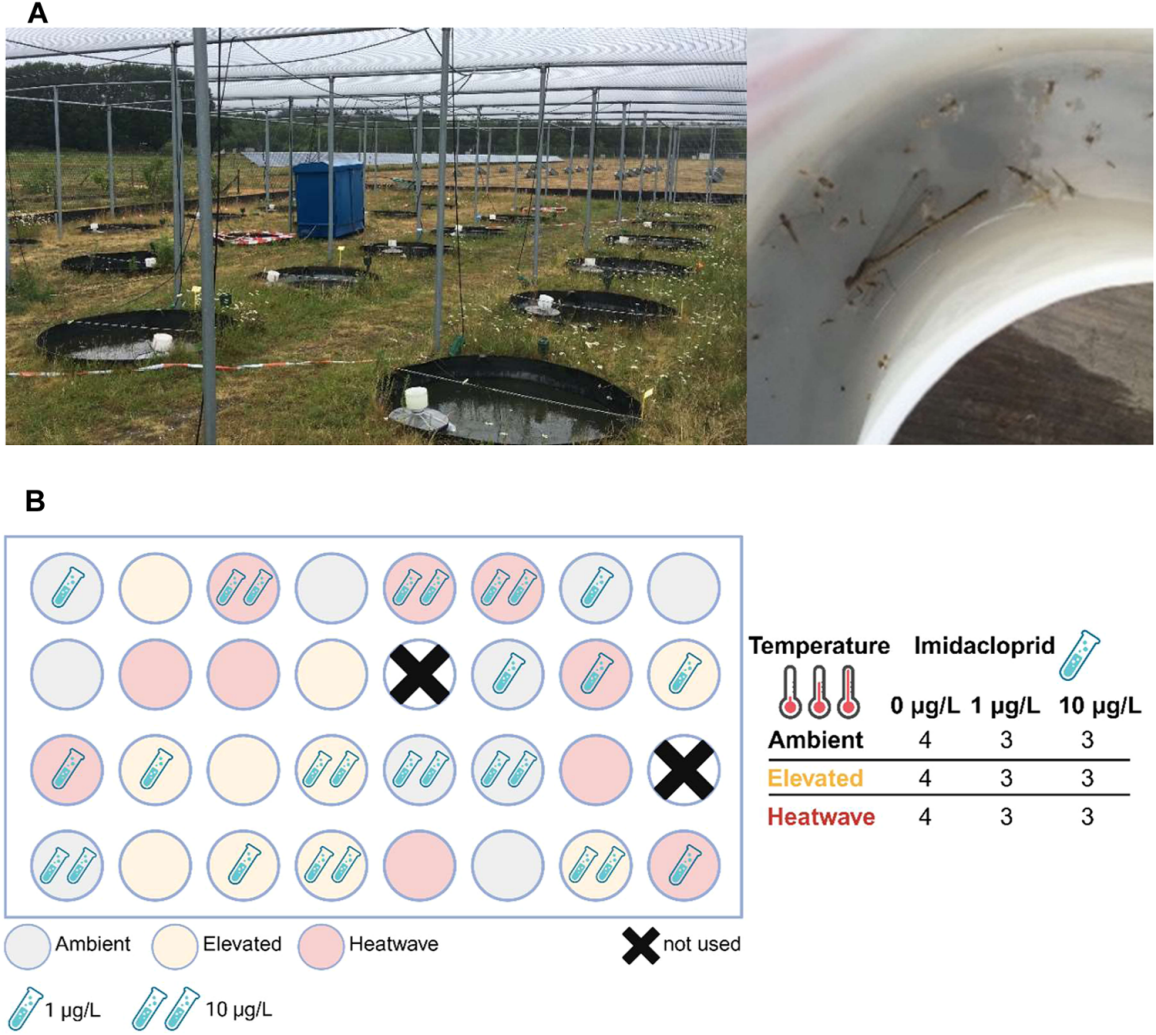
Experimental site with pond mesocosms and emergence traps (A, top left) from which insects were caught in an insect collector box (A, top right). A schematic overview of the experimental field mesocosm experiment (B, bottom left), including ambient (light gray), elevated (orange), and heatwave (red) treatment in combination with imidacloprid (one test tube:1 and two test tubes: 10 μg/L) or as chemical control (no test tube) resulting in four or three replicates for the single and combined treatments, respectively (B, bottom right).

## Materials and Methods

### Research Facility and Aquatic Insect Sampling

The outdoor pond mesocosm experiment took place from June 15th (week 0) to August 21st, 2020 (week 9) at the experimental field station, the Sinderhoeve in Renkum, The Netherlands (51°59′55.2″N 5°45′10.0″E), belonging to Wageningen University & Research (www.sinderhoeve.org). The study period, particularly the beginning of the treatment phase (June 15th to July 27th), was strategically scheduled to minimize the risk of potential interference from natural heatwaves during Central Europe’s summer period (August-September), thereby maintaining environmental relevance to thermal extremes. Additionally, because aquatic insect emergence is critically dependent on water temperature and light availability,^56,57^ starting the experiment earlier would have conflicted with these ecological drivers likely resulting in minimal emergence abundances. Each of the 30 pond mesocosms used was round-shaped (diameter-depth: 1.8–0.8 m; volume: 1270 L) and placed side-by-side at approximately 1.5 m distance from each other (Figure 1B). At the start of a four-month acclimatization period (March to June 2020), all mesocosms contained a 0.2 m layer of silty-sediment and about 1000 L freshwater from a surface water reservoir at the Sinderhoeve. Additionally, all mesocosms were stocked with assemblages of invertebrates, zooplankton, phytoplankton, microbes, and two macrophyte species. All organisms were collected from artificial ditches and other mesocosms present at the Sinderhoeve, as well as from a brook in the vicinity of the field station, resulting in similar experimental pond ecosystems at the start of the experiment. Albeit the mesocosms are hydrologically isolated pond ecosystems and (re)­colonization, considered as a stochastic process, is only feasible for organisms that are mobile in the aquatic and terrestrial realm, they were inhabited by a large number of macroinvertebrates comprising 86 freshwater taxa from several insect orders. More detailed information about the mesocosm setup is provided in ref 55.^55^

### Emergent Insect Traps

Each mesocosm was equipped with one emergence trap which was floating on the water surface to collect emerging insects biweekly over a period of 12 weeks from the end of May until August. Earlier experimental methods to trap emergent insects from aquatic ecosystems involved using small floating tents constructed from polyvinyl chloride (PVC) pipes, covered with a fine white mesh net. These tents included a Velcro opening, allowing insects to be collected from the trap using an aspirator.^58^ Later studies showed that a pyramid-shaped floating insect emergence trap, equipped with an external collection bottle to avoid the need for aspiration, trapped twice as many emerging insects as the tent-style trap.^59^ We used a similar pyramid-shaped emergence trap with a preservative solution in a break-proof insect collector box, considering the reported benefits of insect trapping efficiency (>95% of the emergent abundance) and sampling speed.^59^ Our emergence traps were deployed in their respective mesocosms for 1 week followed by 1 week outside the mesocosm before the next deployment. Each trap was made out of a gray round-shaped base frame (diameter-side height: 40–15 cm) that created a catching surface for emerging insects of 1257 cm^2^. Four empty plastic bottles were fitted with cable ties at the outer wall of each base frame to ensure buoyancy and floating stability. On top of the base frame, a robust pyramidal gauze tent was enclosed and kept in shape by a stainless-steel rod that was screwed at one end to the inner side of the base frame. At the top of each pyramidal trap, a short plastic tube was screwed to the other end of the rod and connected the trap via an opening (diameter: 7.5 cm) to a translucent, round-shaped (diameter-height: 15–11 cm) insect collector box (Figure 1A, right). The pyramid-shaped floating insect emergence trap allowed for nondisruptive sampling of adult insects while insect larvae were undisturbed. The same research staff consistently maintained standardized sampling procedures throughout the study period. During the sampling process, the break-proof plastic insect collector box was closed with a polystyrene lid and filled with 150 mL of solution containing ethanol (70%), saccharose (40 g/L), and glycerin (40 mL/L) that attracts and preserves insects. Following 1 week of sampling, the boxes were emptied and the trapped insects were transferred with the solution to 100 mL polyethylene bottles. All boxes were refilled with a freshly prepared solution before the beginning of the next sampling period.

### Imidacloprid Dosing and Simulation of Heatwaves and Elevated Temperatures

To assess whether insect emergence was similar among all mesocosms, we investigated insect emergence 3 and 1 week prior to the start of the application of the chemical and thermal stressors. Following these 3 weeks, we spiked the mesocosms biweekly between mid-June (week 0) and July (week 4) 2020 with one of the three different concentrations: 0, 1, and 10 μg/L of the imidacloprid formulation Admire (70% active ingredient, Bayer). We chose imidacloprid because of its extensive global use as one of the most widely applied neonicotinoid insecticides with a long application history.^60,33^ Imidacloprid is frequently traced in global surface waters with peak concentrations up to 320 times higher than our lowest applied concentration, posing high acute and chronic risks for surface water ecosystems.^61–63^ Imidacloprid dosing was performed in weeks 0, 2, and 4 using 0.5 and 5 mL of a freshly prepared stock solution (2 g/L in Milli-Q water) in 1 L plastic dosing bottles. The contents of the bottles were evenly distributed over the water surface of the designated mesocosm followed by gently steering. We monitored the imidacloprid concentrations using LC/MS-MS analyses to estimate the required imidacloprid dose for the second and third spike with regard to the nominal target concentrations (see^31^ for chemical analysis). The fate of imidacloprid was evaluated using exponential models based on first-order kinetics for water and log-normal Gaussian distribution models for sediment. Dissipation half-lives (DT_50s_) were derived using the equation:

$${\mathrm{DT}}_{50}=\frac{\ln\left(2\right)}{k}$$

where *k* represents the dissipation rate constant obtained from the least-squares regression of imidacloprid concentration versus time. Time-weighted average concentrations (TWAs) were computed as follows:

$$\mathrm{TWA}=\frac{\sum \left(C_{i}\times \Delta t_{i}\right)}{T}$$

where *C*_*i*_ represents the imidacloprid concentration at time point *i*, Δ*t*_*i*_ is the time interval since the last measurement, and *T* is the total duration of the observation period (see guidance for calculations in Annex 6,^64^). Analytical water and sediment measurements before the first spike demonstrated no detectable imidacloprid concentrations in the mesocosms. The chemical control treatment (*C*_0_) was replicated four times and both chemical treatments (*C*_1_ and *C*_10_) were replicated three times using a full factorial design with the three different climate change-related temperature scenarios: ambient, elevated temperatures, and heatwaves. The “transportable temperature and heatwave control device” (TENTACLE) was used for temperature manipulation and temperature recording in all cosms.^65^ The elevated temperature scenario continuously followed the average temperature measured in the mesocosms with natural ambient temperatures increased by +4 °C. The heatwave scenario reached for 1 week the target temperatures of 29, 27, and 29 °C during the first, second, and third heatwaves, respectively, followed by a decrease to ambient conditions for 1 week and the reoccurrence of the next heatwave. The target temperature of a heatwave was determined by raising the calculated average ambient temperatures measured a few days prior to the next heatwave simulation by +8 °C. The temperature during the heatwave was increased by +8 °C to ensure comparable average energy input (+4 °C) for both temperature scenarios during the treatment period (week 0–6). Heatwave duration was based on average durations of lake heatwaves (7.7 ± 0.4 days,^38^) and extreme events in Western Europe (8.4 days,^37^). While both experimental temperature treatments represent thermal disturbances in aquatic ecosystems, the simulated heatwaves act as repetitive pulsed stressors, marked by abrupt positive and negative temperature alterations over time (“pulse”), while the simulated elevated temperatures act as a progressive stressor (“press”) characterized by gradual temperature changes.^66^

### Insect Identification

The collected insects were first identified to the order level by means of a stereo microscope and European insect identification keys. Subsequently, we further identified all insects to family level and sorted them during the identification process by means of 6-well plates (ThermoFisher Scientific) and Petri dishes. Since insect identification is a labor-intensive process in which more than only one single observer was involved, we decided to identify insects beyond the family level only in a few cases, such as for beetles (Coleoptera). Finally, after insect identification, insects were sorted, pooled together by order, and preserved in 1.5 mL Eppendorf tubes using 80% ethanol.

### Insect Biomass

To determine the biomass of each insect order per treatment and date, all insects were transferred from the Eppendorf tubes to glass microfiber filters (GF/F, diameter: 47 mm, Whatman) and oven-dried for 3 days at 60 °C. Afterward, each microfiber filter containing insects and a blank filter was weighed using a microbalance. The weight of the blank filter was later subtracted from the weight of the filters with insects to calculate the insect biomass. Finally, the weights of the insect orders were summed to obtain the total insect biomass.

### Statistical Analyses

Changes in the emerged insect species composition were analyzed using the principal response curves (PRCs) method using CANOCO V.5.^67–69^ PRC is able to reveal significant effects induced by temperature scenarios, imidacloprid, and their interactions. Prior to the analysis, the insect abundance data was ln (2*x* + 1) transformed (see ref 70 for rationale). The PRC technique is based on redundancy analysis (RDA) and extracts responses of the community composition to the stressors of interest in time from the total variation in the community composition. PRC includes all treatments and their interaction with time as explanatory variables, while sampling week is included as a covariate. Thus, the *y*-axis depicts treatment versus control effects as a regression coefficient (*c*_dt_) against time on the *x*-axis. The affinity of each taxon for the community response is represented by its taxon weight (*b*_k_). A Monte Carlo permutation test by permuting whole time series was used to analyze the overall significance of treatment effects on the variation in community composition shown in the PRC graph. By including only the relevant samples in Monte Carlo permutations under the RDA option, the significance of effects at each isolated sampling week was evaluated.

The significance of the effects of temperature, imidacloprid, sampling week, and their interactions on insect abundances and biomass were investigated using generalized linear mixed models (GLMMs, function “lmer”, package “lme4”^71^) using the ln (2*x* + 1) transformed abundance data and linear regression models programmed in *R* v.4.0.2.^72^ Fixed effects were defined by imidacloprid, temperature, and sampling week (i.e., main effect). Random effects were defined by mesocosm ID to account for the variance between the cosms. To determine the optimal model fit, we compared residual errors (sigma), marginal and conditional *R* ^2^-values, and AIC/BIC values while assessing the contribution of random effects to explained variances. Additionally, we conducted visual inspections of the residual normality using histograms and Q-Q plots. The best-fit model’s parameter estimates were subsequently evaluated via Satterthwaite’s correction method in ANOVA tables, considering F-tests and *p*-values. All graphs were generated using GraphPad Prism v.10 (GraphPad Software).

## Results

### Imidacloprid Concentrations and Water Temperature

Initial imidacloprid concentrations in water were on average 0.0 (control, *C*_0_), 0.9 (nominal concentration 1 μg/L, *C*_1_), and 9.5 μg/L (nominal concentration 10 μg/L, *C*_10_) after the three dosing events across all temperature scenarios. Imidacloprid dissipated relatively fast in all treatments showing average DT_50s_ and time-weighted averages for the 1 and 10 μg/L treatment and three imidacloprid spikes (SI Appendix, Figure S1; Table S1) of 7 and 8 days with 0.3 and 3.8 μg/L under ambient temperatures, 8 and 6 days with 0.3 and 3.3 μg/L under elevated temperatures, and 7 and 6 days with 0.3 and 2.8 μg/L under heatwaves, respectively. Imidacloprid TWA concentrations in the sediment of the high imidacloprid treatment (*C*_10_) were highest under ambient (2 μg/kg), followed by heatwave (1.7 μg/kg) and elevated temperatures (1.6 μg/kg). Imidacloprid in the sediment of the low imidacloprid treatment (*C*_1_) was below the limit of quantification (LOQ < 1 μg/kg).

The ambient water temperature during the experiment ranged from 15.4 to 28.8 °C with average water temperatures during the application phase of 21.2 °C for ambient temperatures, as well as 25.7 and 25.1 °C for the climate change-related temperature scenarios representing elevated temperatures and heatwaves, respectively (SI Appendix, Figure S2).

### Imidacloprid and Temperature Effects on Biomass

The average cumulative insect biomass of both the low and high imidacloprid treatment was significantly lower than the control under elevated temperatures (adj. *R* ^2^ *=* 0.95, *F*_5,12_ = 72.34, *P*_C1_ = 0.032, *P*_C10_ < 0.001; Figure 2A,B,C) and heatwaves (adj. *R* ^2^ *=* 0.94, *F*_5,12_ = 55.90, *P*_C1_ = 0.007, *P*_C10_ = 0.003), while no significant effects of imidacloprid were noted under ambient temperatures. Heatwaves significantly reduced insect biomass in the low imidacloprid treatment (adj. *R* ^2^ *=* 0.95, *F*_5,12_ = 61.74, *P* = 0.024), while no significant effects of heatwaves were recorded in the control or high imidacloprid treatment, nor for the elevated temperature treatment.

**Figure 2:**
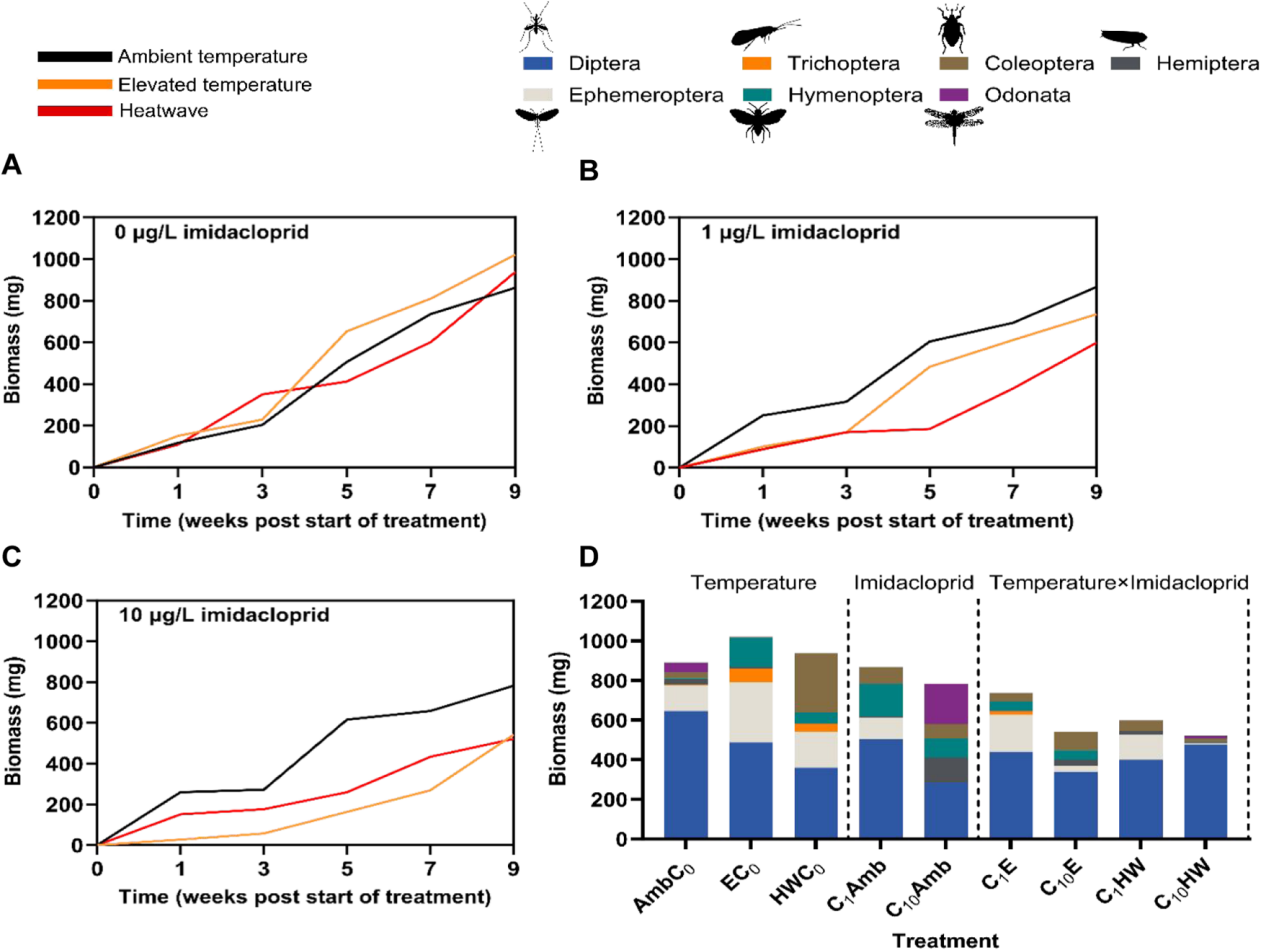
Average cumulative insect biomass throughout the experiment for (A) 0 μg/L, (B) 1 μg/L, and (C) 10 μg/L under the three temperature treatments (ambient, elevated, heatwave), and cumulative total insect biomass (D) at the end of the experiment (week 9) per taxonomic order across the different single and combined treatments (all in mg/1257 cm^2^ water surface with *n* = 4 for chemical controls and *n* = 3 for imidacloprid treatments).

At the end of the experiment (week 9), total cumulative insect biomass was similar and slightly reduced, compared to the chemical control, by 0.2 and 9% in the low and high imidacloprid treatments under ambient temperatures, respectively (Table 1). Under elevated temperatures and heatwaves, total cumulative insect biomass was reduced, compared to the chemical control, by 28 and 47%, as well as by 36 and 44% in the low and high imidacloprid treatment, respectively. Dipterans made up the bulk of the biomass collected at the end of the experiment, comprising on average 48% of the total control biomass, whereas ephemeropterans and coleopterans accounted on average for 19 and 11%, respectively (Table 1; Figure 2D). The remaining biomass (22%) constitutes insects of the orders Hymenoptera, Hemiptera, Odonata, and Trichoptera. Overall, the total biomass of all orders notably decreased under both warming and imidacloprid stress (Figure 2D).

**Table 1:**
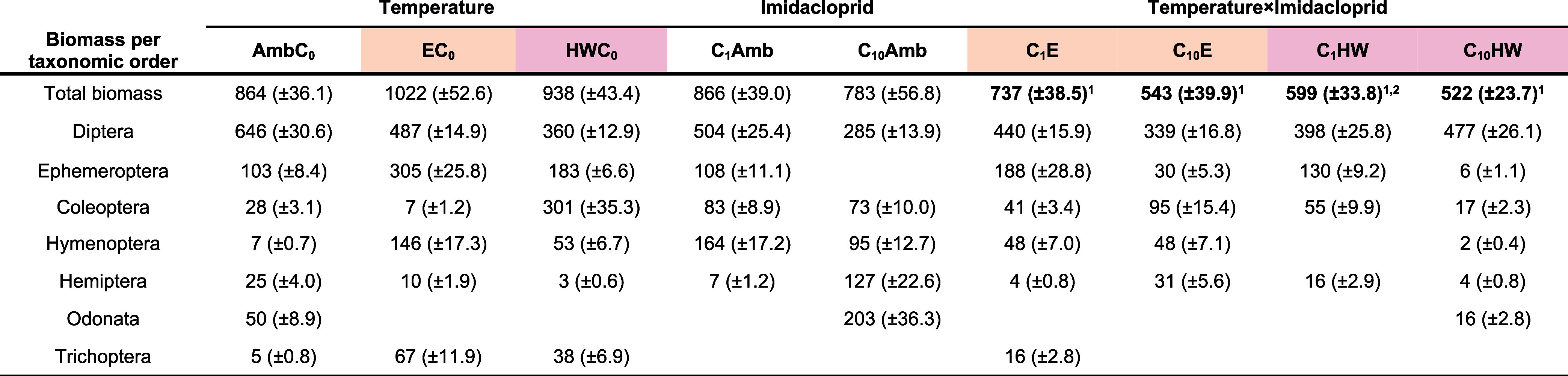
Average Cumulative Total Insect Biomass and Insect Biomass Per Taxonomic Order (in mg/1257 cm^2^ Water Surface (±SE) for *n* = 4 for Chemical Controls and *n* = 3 for Imidacloprid Treatments across the Different Temperature Treatments) at the End (Week 9) of the Experiment for Ambient (Amb), Elevated (E), and Heatwave (HW) Temperatures and 1 μg/L (*C*_1_) and 10 μg/L (*C*_10_) Imidacloprid Treatments^a^

a Empty cells indicate no collected insects. Bold values indicate significance between the chemical treatments and control (Amb*C*_0_, E*C*_0_, and HW*C*_0_) under the same temperature treatment^1^ or between the warming scenarios and ambient conditions under the same imidacloprid treatment^2^.

### Imidacloprid and Temperature Effects on Insect Community Dynamics

We collected 8727 emerged aquatic insect individuals from 74 families across seven insect orders over a 3-month period from the emergence traps. These included 7942 flies and midges (Diptera), 284 mayflies (Ephemeroptera), 159 beetles (Coleoptera), 10 caddisflies (Trichoptera), 3 dragonflies and damselflies (Odonata), 271 hymenopterans (Hymenoptera), and 58 true bugs (Hemiptera). During two sampling dates in the pretreatment period (week −3, −1), 17% of the insects were trapped, whereas 45% were trapped in the three samplings of the treatment period (weeks 1, 3, 5), while 38% were trapped in the two samplings of the post-treatment period (weeks 7, 9). Additional analyses of the macroinvertebrate community at the aquatic larval stage showed nonsignificant differences in community structure prior to the treatment phase, indicating closely similar communities among all treatments (see ref 55 for reference).

The first Principal Response Curve (PRC1, Figure 3A) depicted all seven insect orders with Diptera and Ephemeroptera being most responsive to the individual and combined stressors, i.e., having the highest taxon weights. Diptera was largely dominated by nonbiting midges from the family Chironomidae, whereas Ephemeroptera was primarily dominated by specimens from the family Baetidae. Both insect orders exhibited the highest positive taxon weights (*b*_k_-values, Figure 3A), demonstrating a lower abundance in the imidacloprid treatments compared to the control. The PRC1 analysis revealed that it displays significantly 52% (*p* ≤ 0.001) of the variation in species composition captured by the imidacloprid and different temperature treatments. Of the total variation present, 51% was explained by factor time and 30% by the evaluated treatments, whereas the remaining variance (19%) was due to differences among replicates. The PRC1 graph (Figure 3A) indicated a temperature-dependent separation of the chemical effects, particularly at the end of the experiment from week 5 onward. The results of the Monte Carlo permutation tests supported the displayed treatment-related differences in PRC1 and showed a higher number of significant effects for the low imidacloprid treatment (*C*_1_ vs *C*_0_) for the elevated temperature treatment than for ambient temperatures and heatwaves (Figure 3A, Table 2). The high imidacloprid treatment (*C*_10_) showed a greater series of significant imidacloprid effects under ambient temperatures compared to those at elevated temperatures and heatwaves. Furthermore, the first and third heatwaves induced significant effects on community structure when compared to the ambient control community (Table 2).

**Figure 3:**
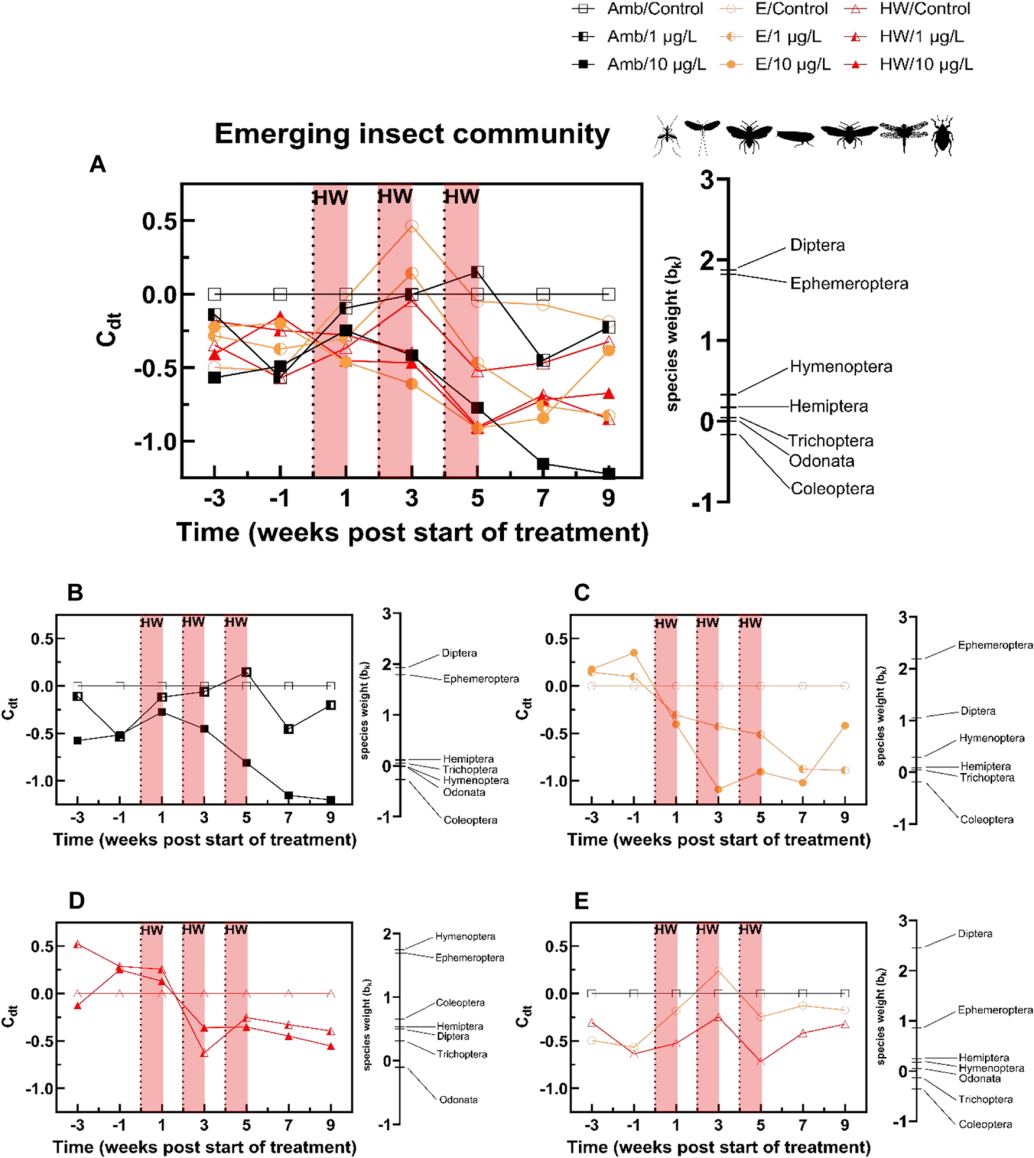
Principal response curves (PRCs) depicting (A) combined temperature and imidacloprid effects, only imidacloprid effects under (B) ambient, (C) elevated, (D) heatwave temperatures, and (E) only temperature effects in the chemical control on the insect community over time. Treatment scores (*c*_dt_) present differences between control and treatments in a sampling week. Insect order-specific affinity to the community response depicted by the PRC is indicated by taxon weights (*b*_k_). The dotted lines indicate the imidacloprid dosing, and the red blocks indicate the timing of the HWs.

**Table 2:**
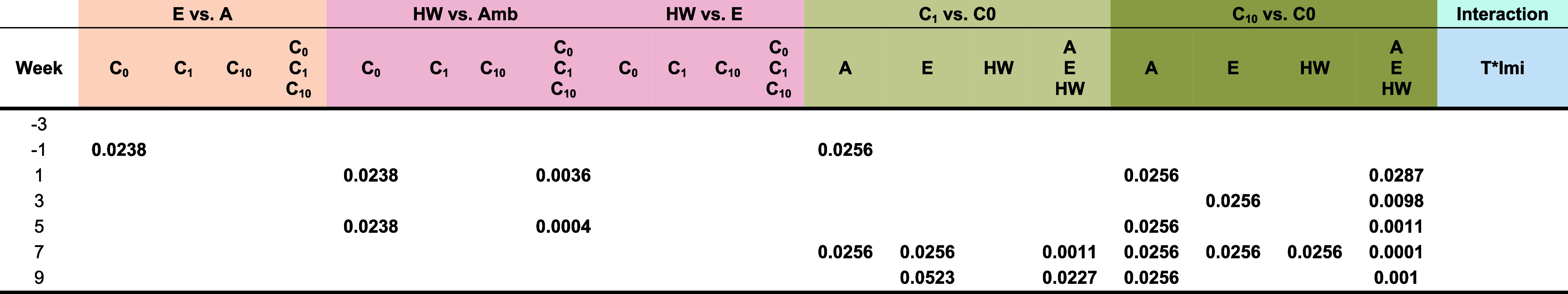
Significant Results (*p*-Values <0.05) from the Monte Carlo Permutations Tests Performed under the Redundancy Analyses (RDA) Option Testing Individual Temperature Effects of Elevated (E) and Heatwave (HW) versus Ambient (A), between HW and E, the Chemical Effects of Imidacloprid (*C*_1_,*C*_10_) versus Control (*C*_0_), and the Interaction of Temperature (T) and Chemical (Imi) on the Insect Community Abundance Structure for Each Sampling Week^a^

a Empty cells denote a *p*-value >0.05.

When analyzed separately, the PRCs of the imidacloprid effects under ambient, elevated, and heatwave temperatures (Figure 3B–D) displayed a significant 71% (*P* = 0.001), 64% (*P* = 0.0045), and a nonsignificant 44% (*P* = 0.5612) of the treatment-related variation in species composition, respectively. The PRC showing only the temperature effects (Figure 3E) displayed a significant 56% (*P* = 0.0162) of the treatment-related variation in species composition. Diptera and Ephemeroptera presented the highest positive taxon weights in the PRCs of the imidacloprid effects under ambient and elevated temperatures as well as for the temperature effects only (Figure 3B,C,E). Hymenoptera and Ephemeroptera exhibited the highest positive taxon weights in the PRC of the imidacloprid effects under heatwaves (Figure 3D).

### Imidacloprid and Temperature Effects on Insect Population Dynamics

The dynamics of the two insect orders showing highest positive taxon weights and total insect abundance toward single and interacting stressors per week are presented in Figure 4. Regarding chemical effects, the high imidacloprid treatment (*C*_10_) caused significantly lower abundances of dipterans and ephemeropterans (Figure 4A,B) compared to the chemical control following the second spike which contributed to the significant effects observed on total insect abundance (Figure 4C). The observed adverse effects on Ephemeroptera persisted until the end of the experiment, while significantly decreased abundances were also noted for the low imidacloprid treatment (*C*_1_) during the last two sampling weeks of the recovery phase (Figure 4B). Regarding thermal effects, only heatwaves induced significantly lower abundances of dipterans, specifically after the first and third heatwave episodes, compared with the ambient temperatures (Figure 4A). Peaks in insect emergence of dipterans appeared after 3 weeks of exposure to elevated temperatures, while a similar peak in emergence was observed after 5 weeks of exposure to ambient temperatures. These effects contributed to the significant temperature effects on total insect abundance (Figure 4C), as dipterans were in the dominant order. Significant stressor interaction effects appeared for Ephemeroptera at the end of the experiment.

**Figure 4:**
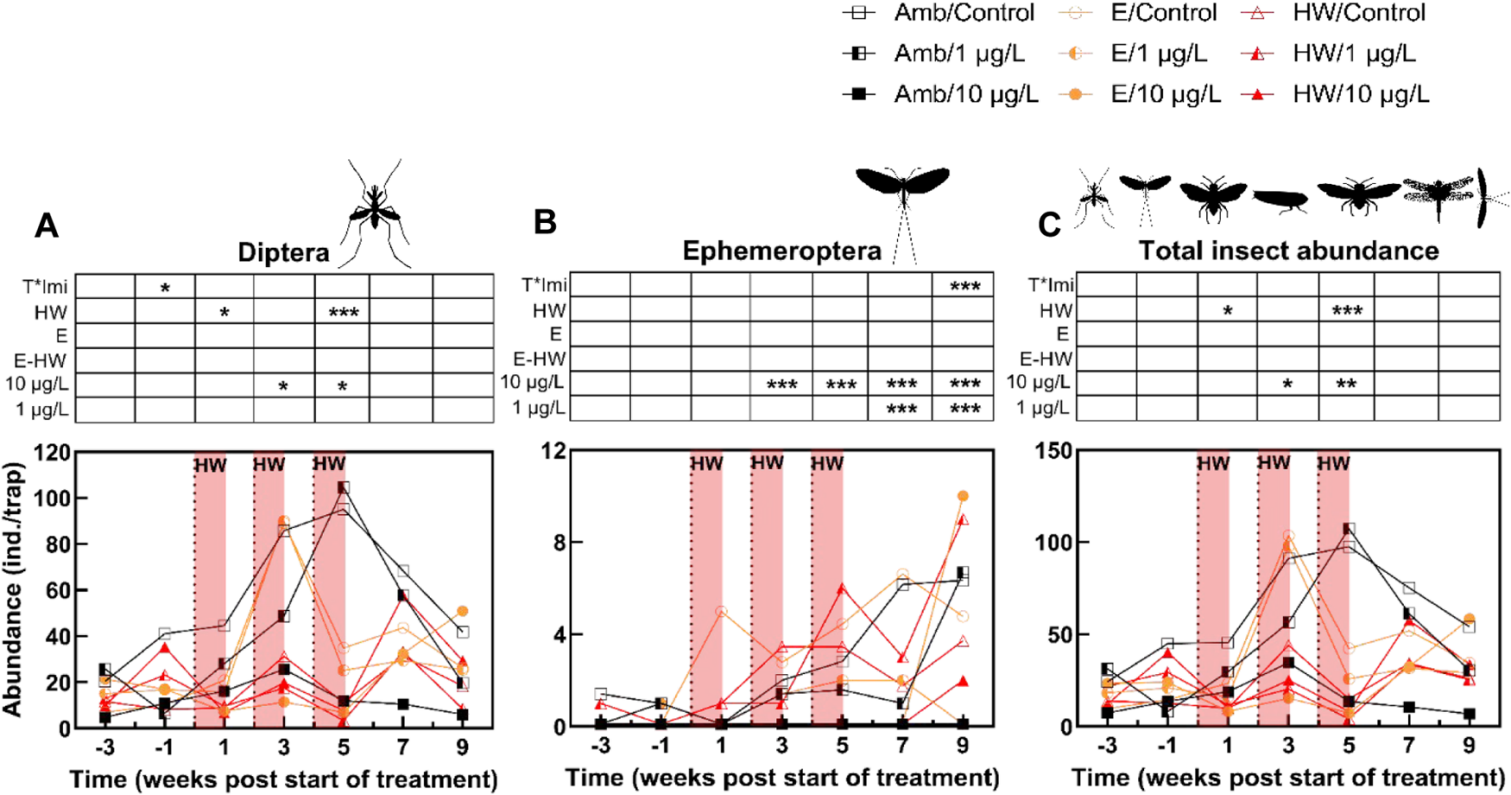
Geometric mean abundance of emerged insects (per trap with 1257 cm^2^ water surface, *n* = 4 for chemical controls and *n* = 3 for imidacloprid treatments across the different temperature treatments) of the most responsive orders (based on the PRC community analyses): (A) Diptera, (B) Ephemeroptera, and (C) all seven insect orders combined. The dotted lines indicate the imidacloprid dosing, and the red blocks indicate the timing of the HWs. Significant effects on abundance of emerged insects for each sampling week and treatment are displayed above the figures, confidence intervals were excluded to facilitate readability.

## Discussion

### Multiple Stressor Effects on Insect Biomass

The observed decline in insect emergence due to the combined exposure to an insecticide and enhanced temperature may not only directly affect freshwater ecosystem function and services but can lead to far-reaching consequences for terrestrial consumers, including insectivorous birds, which rely on aquatic insects as an important source of highly unsaturated omega-3 fatty acids and nutritional subsidies.^73–75^ The high nutritional value of emerging aquatic insects and the importance of pond ecosystems as “insect chimneys” for insectivorous birds are underscored by the positive relationship between the abundance of emergent insects and bird species richness and abundance.^76^ Considering our study results that both elevated temperatures and heatwaves, in combination with the low and high imidacloprid treatments, caused a significant decline of between 28 and 47% in insect biomass (Table 1, Figure 2D), altered food conditions are likely to arise for dependent organisms under ongoing climate change and insecticide application in agricultural landscapes. Although empirical studies on the combined effects of warming and insecticides on aquatic insect biomass are lacking, our results provide evidence that climate warming and pesticides are, in fact, important drivers of the globally observed insect decline.^5^ Furthermore, our results corroborate previous assumptions in ref 77 who considered the effects of synthetic pesticides as a likely driver of the observed decline of 76% in flying insect biomass over 27 years collected at several nature protection sites in Germany. While various environmental factors, such as climate change, barely explained ∼20% of the observed decline in insect biomass in Germany,^77^ our results highlight that both thermal and chemical stressors combined resulted in an almost halving of the emerged insect biomass when compared to control conditions.

### Multiple Stressor Effects on Insect Abundances

The imidacloprid effects on the emergence of aquatic insects largely agree with those of a previous lentic microcosm study where three weekly applied imidacloprid pulses significantly reduced Ephemeroptera emergence at a nominal concentration of 3.2 μg/L and higher (Mean TWA concentration 21d: 1 μg/L;^50^). Moreover, coherent results were reported by a lotic mesocosm study where six imidacloprid pulses of 12 μg/L (TWA concentration of three pulses, 21d: 0.85 μg/L) significantly reduced emergence of predominantly dipterans and ephemeropterans.^49^ Interestingly, both studies imply time-cumulative imidacloprid effects on the emerging insect community at low imidacloprid levels and after three to four pulses (i.e., 8 weeks after the first pulse). This resembles a delayed onset of imidacloprid effects post treatment. In agreement with these two studies, we found for the first time significant effects on the emerging insect community following three imidacloprid pulses at ambient temperatures (i.e., 7 weeks after the first pulse; Figure 3A; Table 2). However, these delayed effects appeared at nominal and average TWA concentrations of 1 and 0.3 μg/L, respectively, which are 3- to 12-fold lower than in the previous studies. Albeit time-cumulative imidacloprid toxicity has been reported in several studies and organisms,^31,78,79^ our observations support the limited but growing body of literature presenting increased imidacloprid toxicity for low levels at chronic exposure.^33,80,81^ Hence, TWA concentrations may be more suitable for comparisons than peak concentrations. The high toxicity of imidacloprid at low levels during chronic exposure can be explained by the biotransformation of the parent compound to imidacloprid-olefin, a toxic metabolite that is eliminated more rapidly from the body of compared to .^31^ The recent finding in ref 82 on the irreversible binding of thiacloprid to nicotinic acetylcholine receptors probably explains the observed lack of elimination of the neonicotinoid in . This resistance may explain the delayed toxic effects of thiacloprid, which may also apply to imidacloprid.

A number of significant effects on the emerging insect community for the low imidacloprid treatment (*C*_1_) were noted at the end of the experiment (Table 2). Other mesocosm studies revealed taxon-specific imidacloprid effects in insect communities,^29,30,52,83^ whereas single-species bioassays demonstrated the highest neonicotinoid sensitivity for mayflies among all tested invertebrates.^31,79^ Acute (4d) and chronic (28d) lethal concentrations (LC_50_) of 26 and 7 μg/L, as well as 0.2 and 0.3 μg/L for and , respectively,^80^ support the field and laboratory observations on mayfly species sensitivity. Our results stress the importance of environmentally realistic outdoor experiments, as they indicate low numbers of emerging ephemeropterans in the heatwave treatment at nominal imidacloprid concentrations considered toxic (1.0 μg/L; Figure 4B). At temperatures lower than those of the heatwave treatment, metamorphosis was presumably unaffected since emergence would otherwise be in much lower numbers. Our observations at these lower temperatures align with those of a recent experimental ditch study with the neonicotinoid thiacloprid where ephemeropterans were thriving at two chemical spikes applied at a nominal concentration of 1.0 μg/L and similar water temperatures (15–30 °C;^46^). On the one hand, our findings imply that ephemeropterans comprises a large bulk of warm-adapted taxa, thus having the capacity to deal with a thermally altered environment and to increase in abundance.^84,85^ On the other hand, higher temperatures may have accelerated development rates of larvae, as it has been reported for , resulting in a shorter life cycle and earlier onset of emergence.^86^ In the latter context, heatwave conditions in our study may have facilitated metamorphosis of ephemeropterans under imidacloprid stress, thus reducing the chemical exposure time.

The high imidacloprid treatment (*C*_10_) induced significant effects on the emerging insect community shortly after the first spike under ambient temperatures, resulting in compositional changes (Table 2, Figure 3A). In the elevated temperatures and heatwave treatments, however, significant structural changes in the emerging insect community appeared later and were significantly less frequent after the second spike and during the recovery phase. The imidacloprid effects under ambient temperatures lasted, except for week 3, until the end of the study, as seen by the deviating course of the chemically stressed community compared to control (Figure 3A). Hence, our results indicate that significant compositional changes of the emerging insect community may appear at relatively low imidacloprid concentrations with regard to globally traced neonicotinoid residues in surface waters of 0.001 to 320 μg/L.^60^

During the treatment phase, total emerging insect abundance in the *C*_10_ treatment was higher under ambient temperatures compared to those at elevated temperatures and heatwaves (Figure 4C). These findings suggest that the susceptibility of aquatic emerging insects to imidacloprid is positively associated with warming, suggesting temperature-enhanced toxicity. Earlier studies on insecticides like imidacloprid, chlorpyrifos, and esfenvalerate provided evidence for increased toxic effects on aquatic species under warming.^53,87–90^ Likewise, observations of more pronounced effects of imidacloprid on mayflies in (sub-) tropical compared to temperate climates^29,80^ corroborate our findings on temperature-enhanced imidacloprid toxicity. It remains questionable though whether the tested populations in the (sub)­tropics were intrinsically more sensitive to temperature or whether the higher temperatures *per se* resulted in higher sensitivity. One possible explanation for the increased species sensitivity to imidacloprid under higher temperatures is the organisms’ elevated uptake rates with increasing imidacloprid biotransformation rates and thus accelerated generation of toxic metabolites, such as imidacloprid-olefin, as reported for .^87,89^ Furthermore, analyses of temperature as modulating factor in toxicokinetic-toxicodynamic (TK-TD) models revealed increasing toxicokinetic rates and altered toxicodynamic parameters under higher temperatures, resulting in increased toxicity under chronic exposures.^88^ The observed inhibition of insect emergence in our study may also arise from imidacloprid-induced impairment of aquatic larval development, following previous observations of disrupted physiological responses in the firefly , including destructive morphological changes, exceeding oxidative stress, and compromised antioxidant defenses under sublethal imidacloprid exposure^91^ which were likely stronger expressed with increased metabolic rates at higher temperatures.

Apart from the high imidacloprid sensitivity of ephemeropterans with a significantly lower emergence irrespective of the temperature treatments (Figure 4B), dipterans appear to be sensitive to imidacloprid, as evidenced by a significantly lower abundance of emerged insects after the second and third spikes in the *C*_10_ treatment, compared to the chemical control (Figure 4A). These findings match with previous observations from a field mesocosm experiment where three imidacloprid pulses at weekly intervals resulted in a significant reduction in the emergence of Chironomidae.^92^ The authors of the study reported a reduced emergence especially at the two highest concentrations of imidacloprid (2.0 and 20 μg/L) with recovery dynamics of the community composition near the end of the study. Conversely to these findings, results from semicontrolled field experiments, of which one used a chronic imidacloprid exposure of 0.5 μg/L (28 days) and the other a weekly repeated doses of up to 0.5 μg/L (9 weeks), demonstrated only subtle effects on the emergence of Chironomidae.^93,94^ In this context, considering the lack of significant effects on dipteran insect emergence at the low imidacloprid concentrations (1 μg/L, TWA: 0.3 μg/L, Figure 4A), it appears that the threshold for negative imidacloprid effects on Diptera emergence may lie within the initial concentration range of 1 to 2 μg/L. This assumption is supported by previous laboratory studies on the emergence of Chironomidae larvae, which revealed lowest observed effect concentrations (LOEC) values of >1.1 and 1.2 μg/L.^48,95^

When the pattern of emerged dipterans over time is compared with the total abundance of emerged insects (Figure 4A,D), it becomes apparent that dipterans have the highest contribution to the total emerged insects. In fact, the dominance of the Chironomidae family within Diptera can be explained by their high occurrence and diversity in freshwater ecosystems.^96^ Furthermore, only Diptera responded significantly to the heatwave treatment, as evidenced by reduced emergence after the first and third heatwaves (Figure 4A), having the heatwave effects on total insect abundance as consequence.^97^ reported in alignment with our findings a significantly reduced number of emerged Chironomidae under warming of +3 °C above an average ambient of 16 °C. Conversely, recent studies investigating heatwave effects on reported only subtle effects on emergence, however, under a temperature scenario of +4 °C versus 20 °C for a period of 2 to 3 days.^98^ The temperature treatment applied in this study aligns well with our elevated temperature treatment, where, indeed, no significant effects on Diptera were noted (Figure 4A). Significantly positive and negative effects of temperature on the total number of emerged insects have been recently reported in heated experimental streams of +3 °C versus an ambient temperature ranging from about 15 to 30 °C.^42^ Depending on the magnitude and duration of the temperature increase, Diptera and Ephemeroptera species, which typically undergo short multivoltine life cycles spanning from a few days to weeks, are likely to either develop faster with smaller imagoes or face population declines when crossing their taxon-specific thermal acclimation zone.^86,99–101^ In fact, differences in the timing of insect emergence were observed with a two-week earlier onset of peaks in total insect emergence under elevated compared to ambient temperatures (Figure 4C). Compared to other physicochemical water quality variables, such as dissolved oxygen concentrations, pH, and nutrients, water temperature proves to be a strong climatic driver shaping insect emergence patterns in aquatic ecosystems as observed in three different lakes in South Germany.^102^ While insects were likely still facing acceptable thermal conditions in the beginning of the treatment phase, thermal thresholds may have been surpassed during the experiment, explaining the sharp increases and decreases in the number of total emerged insects in weeks 1 to 5 (Figure 4C) when temperatures of 30 °C or higher were reached in both temperature treatments (SI Appendix, Figure S2). In addition to the documented temperature effects on insect emergence, significantly higher water conductivity values toward the study’s end under elevated temperature and heatwave (see ref 55 for reference) likely played a contributing role in the observed insect biomass increases (Table 1), mirroring earlier research that identified water conductivity, alongside temperature, as another driver of increased insect biomass.^103^

Overall, our study highlights that the combined effects of warming and insecticide exposure can reduce aquatic insect emergence by nearly 50%, likely causing extensive effects on interconnected terrestrial food webs, ecosystem services, and biodiversity.

## Study Limitations and Future Research

Our study provides empirical evidence of adverse impacts of a neonicotinoid pesticide and climate warming on the life cycle of aquatic insects. However, limitations need to be acknowledged. Our study assessed the effects of chemical and climate change-related stressors in a short-term exposure scenario of 5 weeks (June–July). Still, long-term effects or chronic exposure conditions and potential differences in seasonal effects remain unclear. Furthermore, although our findings are based on local populations and communities, aquatic insect responses may differ across ecoregions because of variations in species composition and environmental conditions. Finally, future experiments could control for potential confounding factors, such as insect immigration, by implementing covers on the experimental pond systems.

By addressing long-term and multigenerational effects throughout different seasons, community responses in different ecoregions, and further development of experimental designs, future research can amend chemical risk assessment and inform conservation strategies to preserve the valuable services and benefits of aquatic insect communities under ongoing climate change.

## Supplementary Material



## Data Availability

The supporting data of this study can be found online at: https://doi.org/10.5281/zenodo.15119468.

## Notes

Environmental fate of imidacloprid in water and sediment (Figure S1, Table S1) and temperature measurements in the aquatic cosms (Figure S2) (PDF)

## †

Present address: (M.H.) Department of Aquatic Ecology, Eawag, Swiss Federal Institute of Aquatic Science and Technology, Dübendorf, Switzerland

## ‡

Present address: (M.K.A.) Toxicology Centre, University of Saskatchewan, 44 Campus Dr, Saskatoon SK S7N 5B3, Canada.
