# Supplementary material for: Multiple Stressor Effects of a Neonicotinoid, Heatwaves, and Elevated Temperatures on Aquatic Insect Emergence
Source: Environ Sci Technol. 2025 Jul 8;59(28):14226–38. doi: 10.1021/acs.est.5c01498 (PMC12288067; doi:10.1021/acs.est.5c01498)
Supplement: Supplementary file 1 [file es5c01498_si_001.pdf]

Supporting information for:

Multiple stressor effects of a neonicotinoid, heatwaves, and elevated temperatures on aquatic insect emergence

Markus Hermann[1,2], Mawuli K. Amekor[1,3], Enzo Contrucci[1],*
Ann M. Evarita[1], Edwin T.H.M. Peeters[1], and Paul J. Van den Brink[1]

[1]Aquatic Ecology and Water Quality Management group, Wageningen University & Research, P.O. Box 47, 6700 AA Wageningen, the Netherlands

Present address:
[2]Department of Aquatic Ecology, Eawag, Swiss Federal Institute of Aquatic Science and Technology, Dübendorf, Switzerland
[3]Toxicology Centre, University of Saskatchewan, 44 Campus Dr, Saskatoon, SK S7N 5B3, Canada
*E-mail: markushermann01@gmail.com, markus.hermann@eawag.ch

This file contains 3 pages including 2 figures and 1 table.
Table of contents:
Figure S1……………………………………………………………………………S2
Figure S2……………………………………………………………………………S3
Table S1……………………………………………………………………………S3

Environmental fate of imidacloprid in water and sediment

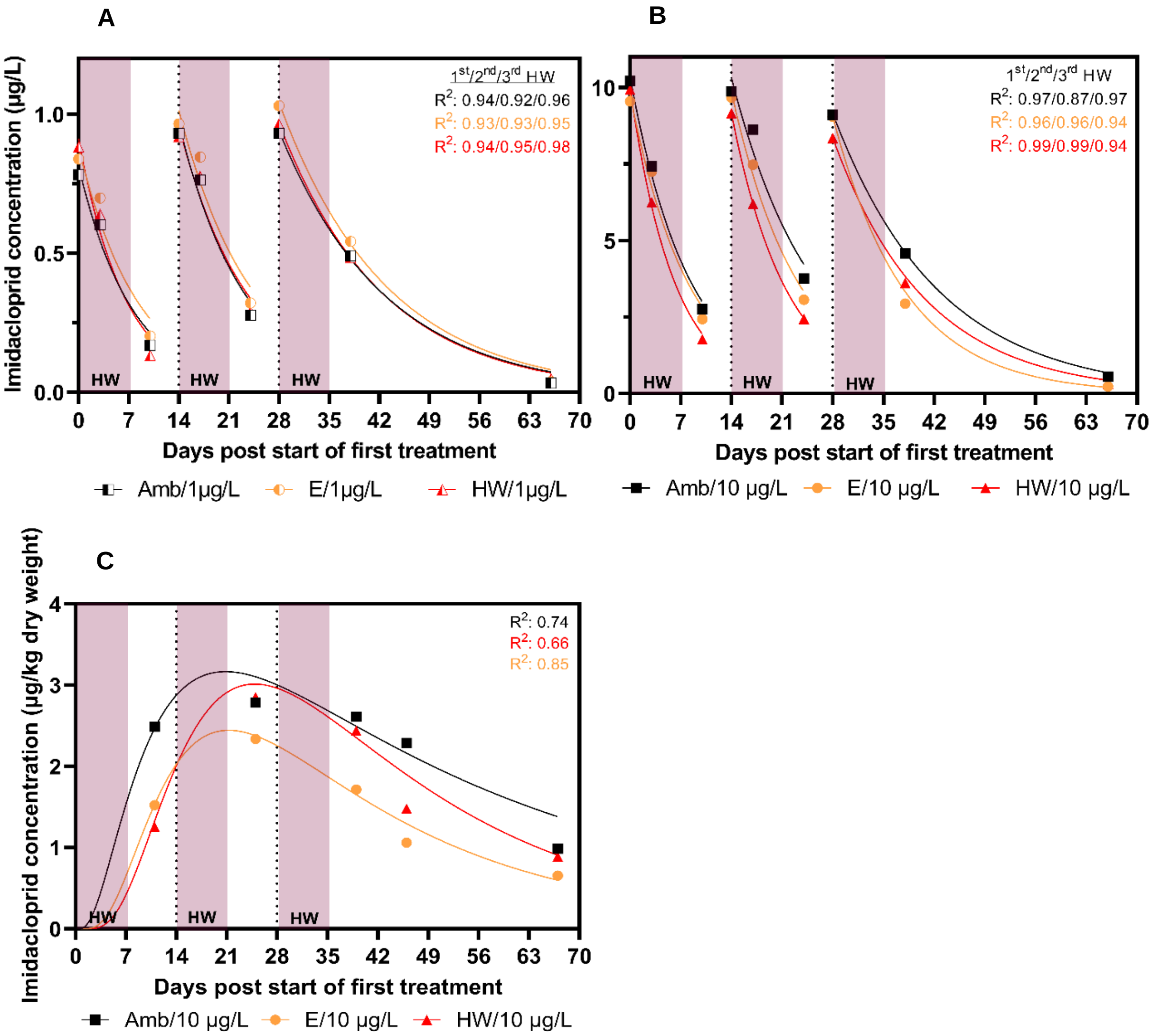

Figure S1. Geometric mean imidacloprid concentrations over 66 days of the experiment of (A) 1 and (B) 10 µg/L in water, and (C) in sediment of 10 µg/L in water under ambient (Amb), elevated (E), and heatwave (HW) temperatures. Red blocks highlight the presence of a heatwave and dotted lines the imidacloprid dosing. Confidence intervals were excluded to facilitate readability. The figure derives from the open access article: *Heatwaves, elevated temperature, and insecticide-induced effects at different trophic levels of a freshwater ecosystem* (Hermann et al. (2025), Ref. 55).

Environmental fate of imidacloprid in water and sediment

Table S1. DT_{50}-values of imidacloprid in water with 95% confidence intervals (C.I.) and time-weighted averages (TWA) in water and sediment (µg/kg dry weight) after the 1st, 2nd, and 3rd imidacloprid dosing (µg/L) and overall, respectively, for the ambient (Amb), elevated (E), and heatwave (HW) treatment. The table derives from the open access article: *Heatwaves, elevated temperature, and insecticide-induced effects at different trophic levels of a freshwater ecosystem* (Hermann et al. (2025), Ref. 55).

		Water						Sediment
		DT_{50} (days, 1 µg/L)	DT_{50} (days, 10 µg/L)	95% C.I. (1 µg/L)	95% C.I. (10 µg/L)	$TWA_{10d,10d,38d}$ (1 µg/L)	$TWA_{10d,10d,38d}$ (10 µg/L)	$TWA_{overall}$
**1st dosing**	Amb_{1st}	**5.19**	**5.58**	**3.83 – 7.27**	**4.61 – 6.83**	**0.32**	**4.18**	**1.95**
	E_{1st}	**5.74**	**5.56**	**4.25 – 8.06**	**4.46 – 7.06**	**0.35**	**3.87**	**1.63**
	HW_{1st}	**4.54**	**4.23**	**3.73 – 6.24**	**3.72 – 4.81**	**0.25**	**3.15**	**1.71**
**2nd dosing**	Amb_{2nd}	**6.36**	**7.75**	**4.61 – 9.23**	**5.28 – 12.61**	**0.45**	**5.27**	
	E_{2nd}	**7.02**	**6.39**	**5.26 – 9.83**	**5.13 – 8.13**	**0.45**	**4.42**	
	HW_{2nd}	**6.64**	**5.27**	**5.19 – 8.76**	**4.76 – 5.86**	**0.45**	**3.54**	
**3rd dosing**	Amb_{3rd}	**10.31**	**10.11**	**7.43 – 14.45**	**7.51 – 13.70**	**0.19**	**2.03**	
	E_{3rd}	**10.33**	**6.92**	**7.34 – 14.70**	**4.48 – 10.80**	**0.23**	**1.48**	
	HW_{3rd}	**10.01**	**8.88**	**7.85 – 12.85**	**5.86 – 13.72**	**0.20**	**1.72**	

Temperature measurements in the aquatic cosms

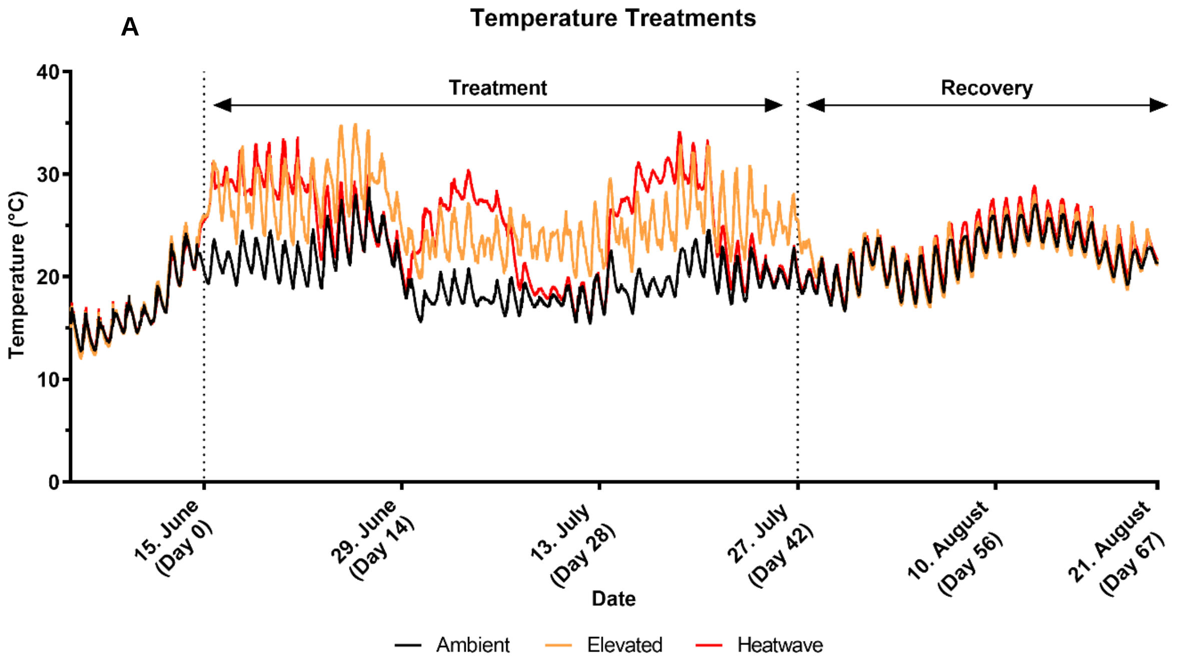

Figure S2. Average temperature recorded in the mesocosm subject to three different temperature scenarios (i.e., ambient (black), elevated (orange), and heatwave (red) in the course of the experiment. The dotted lines indicate the start and end of the treatment phase (i.e., day 0 and day 42) followed by the recovery phase excluding temperature manipulation. The figure derives from the open access article: *Heatwaves, elevated temperature, and insecticide-induced effects at different trophic levels of a freshwater ecosystem* (Hermann et al. (2025), Ref. 55).
